# Serum progesterone levels for ‘rescue’ in hormone replacement FET: a retrospective cohort study of 917 cycles

**DOI:** 10.3389/fendo.2026.1847028

**Published:** 2026-06-01

**Authors:** Goksu Goc, Yavuz Emre Sukur

**Affiliations:** 1 Department of Obstetrics and Gynecology, American Hospital, Prishtine, Kosovo; 2 Department of Obstetrics and Gynecology, Ankara University School of Medicine, Ankara, Türkiye

**Keywords:** assay-specific threshold, frozen embryo transfer, hormone replacement therapy, live birth rate, miscarriage, progesterone, single-dose rescue

## Abstract

**Background:**

In hormone replacement frozen embryo transfer (HRT-FET) cycles with low pre-transfer progesterone, evidence on when single-dose rescue is insufficient is limited. Under an institutional protocol using a 10 ng/mL trigger applied at clinician discretion (no cycles deferred), we describe outcomes and explore whether very low pre-ET P4 identifies a poor-prognosis subgroup, without seeking to establish a deferral threshold.

**Methods:**

Single-center retrospective cohort (2017–2023) of 917 HRT-FET patients grouped by rescue receipt: 650 No-Rescue, 267 Rescue (single 50 mg IM progesterone; all proceeded to transfer). 40 borderline cycles (P4 9.1–9.9 ng/mL) were managed without rescue at clinician discretion. Progesterone was measured by Cobas^®^ e411 ECLIA. The Rescue group was stratified by pre-ET P4 (<5, 5–7.49, 7.5–9.99 ng/mL). Analyses included Fisher’s exact, multivariable logistic regression, restricted cubic splines, ROC, and 1000-resample bootstrap.

**Results:**

Within the Rescue group, LBR was lower when pre-ET P4 was <5 ng/mL: 19.2% (n=78) versus 43.8% (5–7.49, n=112) and 39.0% (7.5–9.99, n=77; trend p=0.010). The <5 vs. ≥5 comparison yielded OR 0.33 (95% CI 0.17–0.62; p=0.0004); aOR 0.35 (95% CI 0.18–0.66; p=0.002). Miscarriage among clinical pregnancies was 46.7% below 5 versus 9.9% above. Rescue with P4 ≥5 had unadjusted LBR similar to No-Rescue (41.8% vs. 39.2%; p=0.55), without isolating a rescue effect. Among 61 borderline cycles (9.1–9.9 ng/mL), LBR was numerically higher with rescue (57.1%) than without (40.0%; p=0.28), but underpowered. ROC AUC was 0.609 (95% CI 0.54–0.68); Youden cutoff 5.3 ng/mL. In 1000 bootstrap resamples, the median Youden threshold was 5.4 ng/mL (IQR 5.0–5.6).

**Conclusions:**

Under a single 50 mg IM rescue protocol, pre-ET P4 <5 ng/mL was associated with poor per-transfer prognosis (LBR ~19%, miscarriage ~47%). This is an exploratory, assay-specific (Cobas e411 ECLIA) signal, not a validated deferral rule, and requires recalibration for other platforms. Because no cycles were deferred and the only rescue-versus-no-rescue contrast was confined to a small 9.1–9.9 ng/mL borderline subset, the rescue effect cannot be formally isolated. Shared decision-making is advised for P4 <5 ng/mL; external validation is required.

## 1 Introduction

In programmed hormone replacement therapy, frozen embryo transfer (HRT-FET), exogenous estrogen and progesterone create an artificial implantation window. Peri-transfer serum progesterone has emerged as a critical determinant of success, with multiple studies demonstrating reduced pregnancy and live birth rates when levels are suboptimal (1–5, 22). Inadequate progesterone impairs endometrial decidualization, disrupts progesterone-dependent gene expression critical for implantation receptivity, and compromises the immune modulation necessary for embryo tolerance (6, 7).

When pre-transfer progesterone is low, clinicians face a practical dilemma: supplement with rescue progesterone and proceed, intensify luteal support, or defer transfer. Rescue strategies vary by route, dose, and duration. Some centers have used a single intramuscular (IM) injection empirically, whereas recent evidence, including Le et al. (2025), supports repeated daily IM supplementation in low-progesterone HRT-FET cycles (8, 15, 23). Because dose frequency can change exposure during the implantation window, findings from single-dose rescue protocols should be distinguished from daily-dose regimens.

Previous studies have reported associations between low progesterone and reduced outcomes (9–11) but most evaluated non-rescued populations or rescue-initiation thresholds rather than the prognosis after a specific rescue protocol has already been administered. Lopez Marin et al. (2025) reported impaired outcomes among patients with very low P4 despite individualized luteal support (16), supporting the need to distinguish rescue initiation, rescue intensification, and post-rescue poor-prognosis thresholds.

We therefore reframed the objective as exploratory and protocol-specific. We aimed to: (1) describe reproductive outcomes in patients managed with the institutional single-dose IM rescue protocol versus those not requiring rescue; (2) explore whether very low pre-ET progesterone within the Rescue group was associated with lower LBR or higher miscarriage; (3) descriptively compare outcomes above and below a candidate low-P4 cutoff with No-Rescue cycles, while recognizing that this comparison cannot isolate the causal effect of rescue; and (4) evaluate perinatal safety among live births. This study was not designed to prove that rescue is effective or that cycle deferral improves outcomes.

## 2 Materials and methods

### 2.1 Study design and ethical approval

This retrospective cohort study was conducted at the American Hospital, Pristina, Kosovo, and included all HRT-FET cycles performed between January 2017 and December 2023. The rescue protocol (50 mg IM progesterone, 10 ng/mL trigger threshold), assay platform, and clinical decision-making framework remained unchanged throughout the study period. The study was approved by the Kosovo Doctors’ Chamber Ethics Committee (Ref. 153/2025), and informed consent was waived by the committee due to the retrospective design. The study follows STROBE guidelines (12); the completed checklist is provided as **Supplementary Table S1**.

### 2.2 Participants

Inclusion criteria: (1) programmed HRT-FET cycle, (2) serial progesterone monitoring at pre-transfer, transfer-day, and post-transfer timepoints, (3) complete outcome data. Each patient contributed one cycle (first eligible); 43 patients had multiple cycles during the study period, of which only the first was included. Cycles that did not meet endometrial criteria (≥7 mm with trilaminar morphology) were canceled before progesterone monitoring and are not included. Cycles canceled before progesterone monitoring due to inadequate endometrial development may differ systematically from included cycles (e.g., more severe endometrial pathology, different patient characteristics). If so, this could limit generalizability to patients with optimal endometrial response. The final cohort comprised 917 unique patients after data quality verification (Section 2.7).

### 2.3 Endometrial preparation protocol

All cycles used a programmed HRT protocol. Endometrial preparation employed oral estradiol valerate in 96.0% of cycles, with minorities receiving patches (1.6%), vaginal (1.3%), or transdermal estradiol (1.1%). Progesterone supplementation was initiated when endometrial thickness reached ≥7 mm and trilaminar morphology was present. The regimen comprised vaginal progesterone gel (82.9%) or micronized vaginal progesterone (17.1%). Embryo transfer was scheduled after at least 5 days of progesterone exposure for blastocyst transfers.

### 2.4 Progesterone monitoring and rescue protocol

Serum progesterone was quantified at three timepoints: (i) the day before ET (pre-ET; 08:00–10:00), (ii) the day of ET (pre-procedure; 08:00–11:00), and (iii) ET + 5 (08:00–10:00). For No-Rescue patients, all three samples were trough draws (≥24 h since the last vaginal progesterone dose). For Rescue patients, the pre-ET and ET + 5 draws were similarly trough samples relative to the vaginal regimen; however, the ET-day draw was obtained approximately 12–14 hours after the IM rescue injection and therefore falls on the descending limb of the IM pharmacokinetic curve (peak typically 6–8 h), representing neither a true trough nor a peak. Samples were analyzed using an electrochemiluminescence immunoassay (ECLIA) on a Cobas^®^ e411 analyzer with Elecsys^®^ Progesterone III reagents (Roche Diagnostics, Mannheim, Germany), which is traceable via isotope dilution GC-MS to a highly purified progesterone standard. The analytical measuring range was 0.030–60 ng/mL, with intra- and interassay coefficients of variation <5%. Results are reported in ng/mL (to convert to nmol/L, multiply by 3.18). Multiple reagent lots were used over the 7-year study period; calibration was verified per the manufacturer’s schedule and internal quality control procedures.

The institutional protocol used a 10 ng/mL pre-ET threshold to trigger rescue. Patients who received rescue (n=267) were given a single 50 mg IM injection of progesterone on the evening of the pre-ET blood draw and constitute the Rescue group; the baseline vaginal regimen was continued unchanged. Patients who did not receive rescue (n=650) constitute the No-Rescue group. The rescue criterion was applied at clinician discretion: cycles with P4 <9.1 ng/mL uniformly received rescue, cycles with P4 ≥10 ng/mL uniformly did not, and 61 cycles in the borderline 9.1–9.9 ng/mL window were managed with rescue (n=21) or without (n=40) according to the treating clinician’s judgment. The 40 borderline cycles managed without rescue are included in the No-Rescue group; their outcomes versus those of borderline cycles that received rescue are reported as a small naturalistic sensitivity comparison (Section 3.5). No cycles were deferred during the study period; all 917 patients proceeded to embryo transfer. Consequently, the study contains no randomized rescue versus no-rescue comparison and cannot estimate either the causal effect of rescue or the potential benefit of deferral.

### 2.5 Outcome definitions

The primary outcome was live birth rate (LBR) per embryo transfer. Secondary outcomes included: clinical pregnancy rate (CPR; intrauterine gestational sac with fetal cardiac activity at 6–7 weeks; biochemical pregnancies were excluded); miscarriage rate per clinical pregnancy (pregnancy loss between clinical pregnancy confirmation and 24 weeks); and stillbirth (fetal loss ≥24 weeks; one case at 32 weeks, classified separately from miscarriage and excluded from live-birth perinatal analyses). Perinatal outcomes included gestational age, birth weight, preterm birth (re-derived as GA <37 weeks), small for gestational age (SGA, <10th percentile), NICU admission, gestational diabetes mellitus (GDM), hypertensive disorders, and mode of delivery. Perinatal outcomes were analyzed only among live births.

### 2.6 Exposure and stratification

Patients were classified into No-Rescue (did not receive IM rescue, n=650) and Rescue (received single-dose 50 mg IM rescue, n=267). The Rescue group was stratified into three exploratory subgroups by pre-ET P4: <5.0, 5.0–7.49, and 7.50–9.99 ng/mL. These categories were selected to describe the low-P4 range and to examine a candidate <5 ng/mL poor-prognosis signal; the cutoff was not prespecified as a clinical deferral rule. The Rescue group was further dichotomized as <5 (n=78) and ≥5 (n=189) for exploratory analyses.

### 2.7 Data quality

Prior to analysis, all variables in the analytic dataset were re-checked against the source patient records to ensure data quality. Records with internal inconsistencies were verified against patient files and corrected to match the source documentation. Biological consistency checks were repeated after correction. Residual undetected errors cannot be excluded and are acknowledged as a limitation.

### 2.8 Statistical analysis

Continuous variables are presented as mean ± SD and compared using independent t-tests or Mann–Whitney U tests. Categorical variables are presented as n (%) with chi-square or Fisher’s exact tests. Cochran–Armitage test assessed dose–response trends. Complete-case analysis was used throughout. Because this was an exploratory retrospective analysis, p values were interpreted descriptively and no single test was considered confirmatory.

Multivariable logistic regression estimated adjusted odds ratios (aORs) for the binary candidate cutoff (<5 vs. ≥5 ng/mL), adjusting for age, BMI, AMH, number of embryos transferred, progesterone formulation, endometrial thickness, and embryo morphological quality. ΔP4 (pre-ET to ET-day change) was not included in the adjusted models because it is a post-rescue variable that may represent part of the pharmacokinetic response to the intervention rather than a baseline confounder; an exploratory continuous-P4 model used the same baseline covariates without ΔP4 adjustment. Variance inflation factors (VIFs>5 considered problematic) assessed multicollinearity; Hosmer–Lemeshow test assessed calibration; the C-statistic assessed discrimination. To evaluate post-rescue progesterone kinetics, ET-day to ET + 5 changes were formally assessed using paired t-tests within groups and a Welch test for the between-group difference in change. Within the Rescue group, ET + 5 progesterone was also compared between pre-ET <5 and ≥5 ng/mL subgroups (Welch test) and was evaluated as an exploratory post-transfer predictor of LBR using univariable logistic regression; these post-transfer analyses are descriptive given the observational design.

Restricted cubic splines (RCS) with 4 knots (at the 5th, 35th, 65th, and 95th percentiles) were used to model the nonlinear P4–LBR relationship. ROC analysis assessed discriminative ability; Youden’s J identified the optimal data-driven cutoff. To examine internal stability, we performed 1000 nonparametric bootstrap resamples within the Rescue group; in each resample, AUC and the Youden cutoff were estimated, and cutoff performance was evaluated in out-of-bag observations. Sensitivity analyses included blastocyst-only and single-embryo-transfer (SET) subgroups, and a small naturalistic comparison of borderline (P4 9.1–9.9 ng/mL) cycles managed with versus without rescue. We did not adjust for multiple comparisons because the statistical approaches were used as convergent descriptive analyses for one core exploratory association rather than as independent confirmatory tests; the cutoff is not treated as clinically actionable without external validation. All tests were two-sided and p values are reported descriptively rather than against a fixed significance threshold. Analyses were conducted in R 4.5.2 using the packages rms, pROC, broom, and car (**Supplementary Table S2**).

## 3 Results

### 3.1 Study population

**Figure 1:**
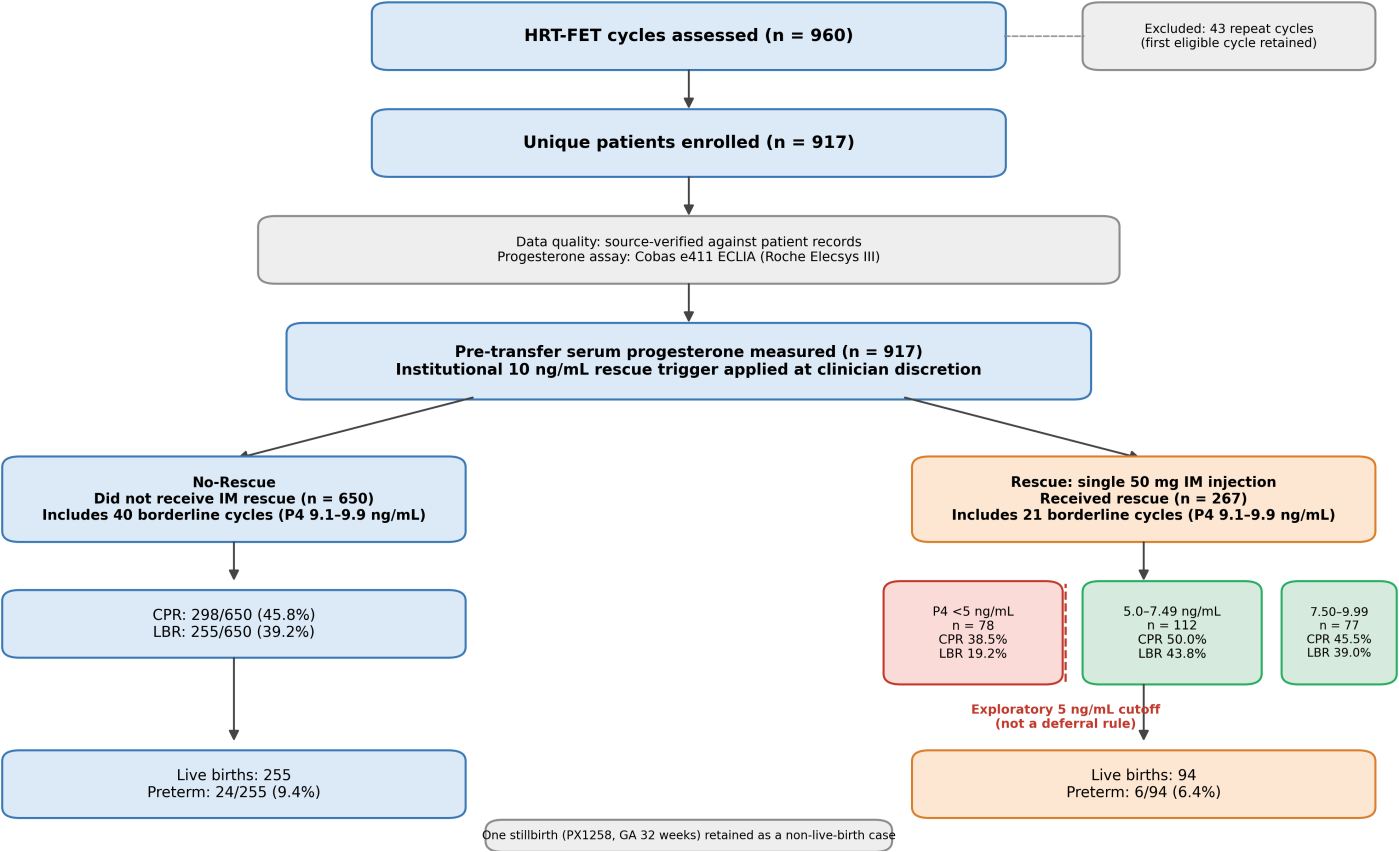
Patient flow and group allocation. No-Rescue denotes cycles that did not receive IM rescue; 40 borderline cycles with pre-ET P4 9.1–9.9 ng/mL managed without rescue at clinician discretion are included in this group.

The cohort comprised 917 unique patients: 650 No-Rescue (70.9%) and 267 Rescue (29.1%; **Figure 1**). Baseline characteristics were broadly balanced, with most SMDs <0.1; infertility type (SMD 0.186) and pre-transfer P4 differed by design (**Table 1**).

**Table 1:** Baseline demographic and clinical characteristics by study group.

Characteristic	No-rescue (n=650)	Rescue (n=267)	p	SMD
Participants, n	650	267		
Age, years	31.98 (6.22)	31.27 (6.14)	0.114	0.115
BMI, kg/m²	27.93 (7.57)	27.50 (7.10)	0.429	0.058
Gravidity	0.00 [0.00, 1.00]	0.00 [0.00, 0.00]	0.171	0.086
Parity	0.00 [0.00, 0.00]	0.00 [0.00, 0.00]	0.429	0.075
Smoking status — Never	587 (90.3)	247 (92.5)	0.325	0.117
Smoking status — Former	42 (6.5)	16 (6.0)		
Smoking status — Current	21 (3.2)	4 (1.5)		
Duration of infertility, years	7.43 (1.61)	7.58 (1.96)	0.214	0.087
Infertility type — Primary	534 (82.2)	199 (74.5)	0.011	0.186
Infertility type — Secondary	116 (17.8)	68 (25.5)		
FSH, IU/L	6.52 (1.36)	6.70 (1.41)	0.081	0.126
LH, IU/L	7.21 (1.79)	7.45 (1.87)	0.070	0.131
Estradiol, pg/mL	39.70 [34.80, 44.48]	40.20 [35.00, 45.25]	0.427	0.072
AMH, ng/mL	2.80 [1.60, 6.00]	2.80 [1.80, 4.90]	0.744	0.030
Previous IVF cycles	2.17 (1.07)	2.30 (1.11)	0.113	0.114
Oocytes retrieved	10.08 (3.39)	10.13 (3.08)	0.807	0.017
Mature oocytes (MII)	6.87 (3.93)	7.06 (3.70)	0.491	0.049
Fertilized oocytes (2PN)	5.06 (2.86)	5.42 (3.07)	0.102	0.123
Blastocysts formed	3.07 (2.14)	3.30 (2.38)	0.178	0.102
Embryos transferred	1.16 (0.37)	1.19 (0.39)	0.308	0.073
Transfer type — SET	544 (83.7)	216 (80.9)	0.356	0.073
Transfer type — DET	106 (16.3)	51 (19.1)		
Embryo stage transferred — Blastocyst	606 (93.2)	247 (92.5)	0.805	0.028
Embryo stage transferred — Cleavage	44 (6.8)	20 (7.5)		
Embryo quality — Good (AA)	209 (32.2)	100 (37.5)	0.263	0.119
Embryo quality — Average (AB/BA)	217 (33.4)	78 (29.2)		
Embryo quality — Fair/Other	224 (34.5)	89 (33.3)		
Progesterone formulation — Micronized progesterone	108 (16.6)	50 (18.7)	0.501	0.055
Progesterone formulation — Vaginal progesterone gel	542 (83.4)	217 (81.3)		
Endometrial thickness, mm	9.05 (1.29)	8.95 (1.31)	0.305	0.074
Pre-transfer progesterone, ng/mL	13.84 (2.87)	6.07 (2.13)	<.001	3.071

Values are shown as mean (SD), median [IQR], or n (%). SMD indicates standardized mean difference.

### 3.2 Serial progesterone trajectories

Rescue patients had clinically similar ET-day progesterone levels to No-Rescue patients (16.4 ± 4.3 vs. 17.0 ± 4.2 ng/mL), although the Rescue ET-day value reflects a post-injection, non-trough sample obtained approximately 12–14 hours after the IM injection and should not be interpreted as sustained luteal exposure. By ET + 5, mean progesterone was 7.8 ± 3.1 ng/mL in Rescue versus 14.0 ± 6.2 ng/mL in No-Rescue (**Table 2**). Formal paired-trajectory analysis confirmed a substantially larger post-injection decline in the Rescue group: ET-day to ET + 5 change was −8.59 ± 5.20 ng/mL (95% CI −9.22 to −7.96; paired p<0.001) versus −3.09 ± 7.08 ng/mL (95% CI −3.64 to −2.54) in No-Rescue; the between-group difference in change was −5.50 ng/mL (95% CI −6.33 to −4.67; Welch p<0.001). Within the Rescue group, however, ET + 5 progesterone did not differ between pre-ET <5 and ≥5 ng/mL subgroups (7.71 ± 2.92 vs. 7.86 ± 3.20 ng/mL; Welch p=0.72), and ET + 5 progesterone was not associated with LBR in exploratory univariable logistic regression (OR per 1 ng/mL 0.98; 95% CI 0.90–1.06; p=0.57). For Rescue patients, pre-ET and ET + 5 were trough samples relative to the vaginal regimen.

**Table 2:** Serial serum progesterone levels by group and timepoint.

Group	Pre-ET P4, ng/mL	ET-day P4, ng/mL	ET+5 P4, ng/mL	Pregnancy-test P4, ng/mL	ΔP4 (ET-day – pre-ET), ng/mL
No-Rescue	13.84 (2.87)	17.05 (4.21)	13.96 (6.16)	15.79 (7.75)	3.21 (4.84)
Rescue	6.07 (2.13)	16.40 (4.30)	7.81 (3.11)	9.66 (6.23)	10.33 (4.56)

Values are mean (SD). For No-Rescue patients, all measurements were trough relative to the vaginal regimen. For Rescue patients, pre-ET and ET + 5 were trough relative to vaginal progesterone; ET-day P4 was measured approximately 12–14 h after the single 50 mg IM rescue dose and is not a trough value. ΔP4 denotes ET-day progesterone minus pre-ET progesterone. ΔP4 is reported descriptively and was not used as an adjustment covariate in the multivariable models.

### 3.3 Primary outcomes: rescue versus no-rescue

CPR was comparable: 45.8% (No-Rescue) versus 45.3% (Rescue; p=0.94). LBR was numerically lower in the Rescue group (35.2% vs. 39.2%) but did not reach significance (p=0.29). Miscarriage per clinical pregnancy was 19.0% (Rescue) versus 14.4% (No-Rescue; Fisher p=0.26; **Table 3**). In multivariable analysis, rescue was not independently associated with LBR (aOR 0.84; 95% CI 0.62–1.13; p=0.24). The only significant predictor was the number of embryos transferred (aOR 1.52; 1.03–2.24; p=0.03). Model C-statistic was 0.569; Hosmer–Lemeshow p=0.81; all non-intercept VIFs <1.5. Subgroup analyses within the Rescue group are reported below.

**Table 3:** Primary reproductive outcomes by study group.

Group	Clinical pregnancy, n/N (%) [95% CI]	Live birth, n/N (%) [95% CI]	Miscarriage per clinical pregnancy, n/N (%)
No-Rescue	298/650 (45.8%; 42.0–49.8)	255/650 (39.2%; 35.5–43.1)	43/298 (14.4%)
Rescue	121/267 (45.3%; 39.2–51.5)	94/267 (35.2%; 29.5–41.3)	23/121 (19.0%)

CPR indicates clinical pregnancy rate; LBR, live birth rate. Exact binomial 95% confidence intervals are shown for CPR and LBR.

### 3.4 Exploratory progesterone subgroup analysis within the rescue group

**Figure 2:**
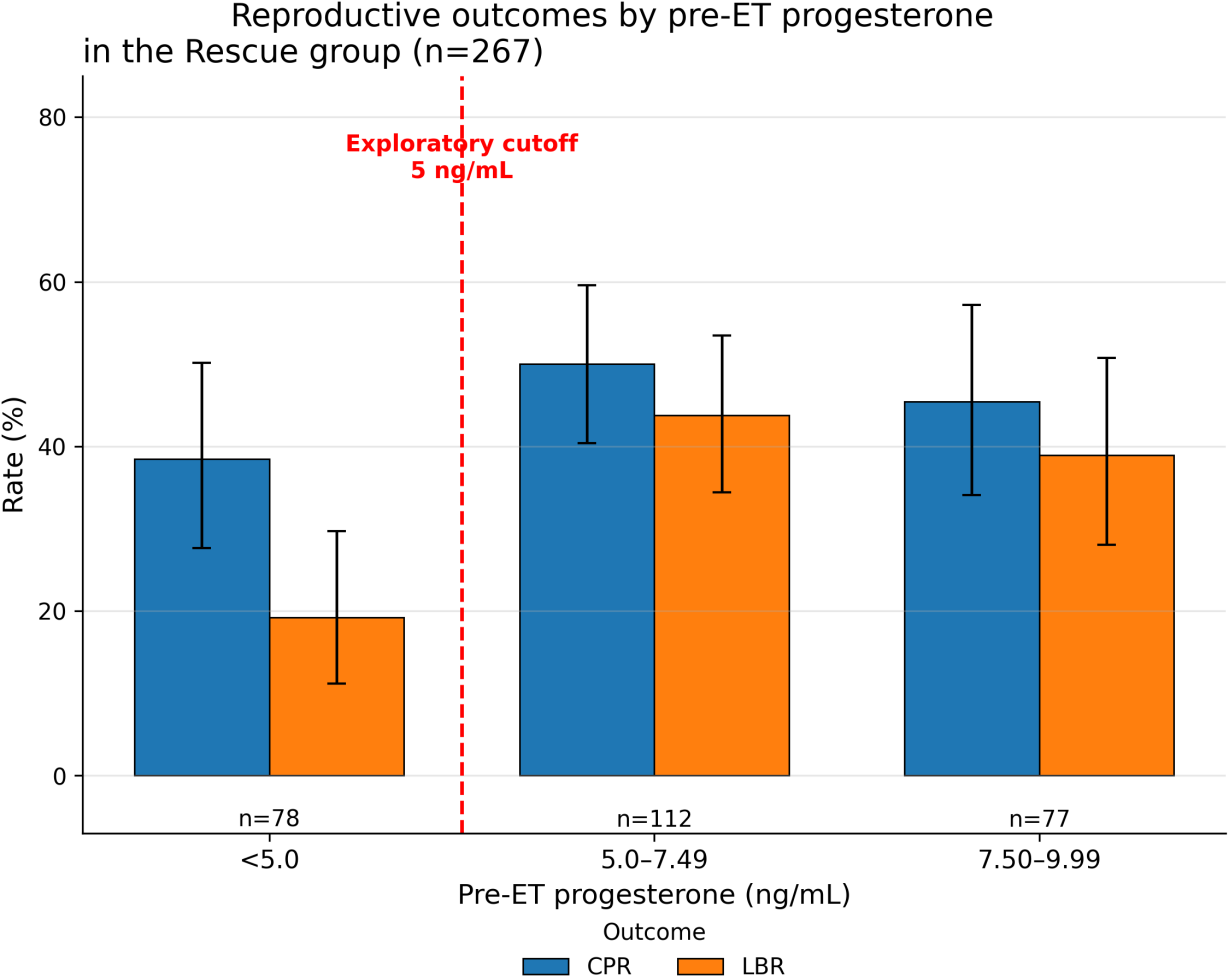
Clinical pregnancy rate and live birth rate by pre-transfer progesterone subgroup within the rescue group. The dashed red line marks the exploratory 5 ng/mL candidate cutoff.

Within 267 Rescue patients, LBR varied across pre-ET P4 strata: 39.0% (7.50–9.99 ng/mL, n=77), 43.8% (5.0–7.49, n=112), and 19.2% (<5.0, n=78; Cochran–Armitage trend p=0.010; **Figure 2**; **Table 4**).

**Table 4:** Reproductive outcomes by pre-transfer progesterone subgroup within the Rescue group.

Rescue subgroup	n	Clinical pregnancy, n/N (%) [95% CI]	Live birth, n/N (%) [95% CI]	Miscarriage per clinical pregnancy (%)
<5.0	78	30/78 (38.5%; 27.7–50.2)	15/78 (19.2%; 11.2–29.7)	46.7
5.0–7.49	112	56/112 (50.0%; 40.4–59.6)	49/112 (43.8%; 34.4–53.4)	12.5
7.50–9.99	77	35/77 (45.5%; 34.1–57.2)	30/77 (39.0%; 28.0–50.8)	5.7

Cochran–Armitage trend test for live birth across ordered subgroups: p = 0.010.

**Figure 3:**
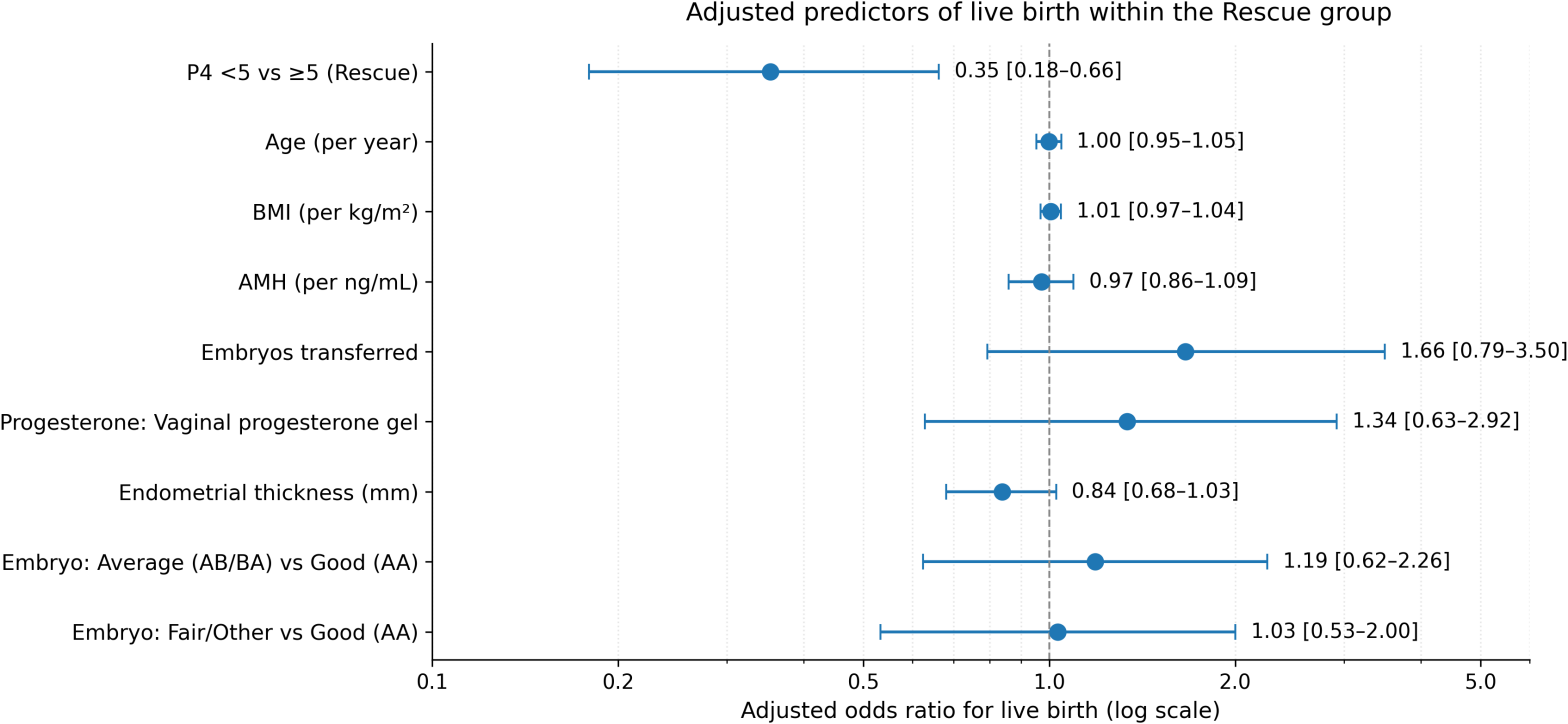
Forest plot of adjusted odds ratios for live birth within the rescue group (<5 vs. ≥5 ng/mL and covariates).

Using the exploratory binary cutoff, LBR was 19.2% (15/78) below 5 ng/mL versus 41.8% (79/189) at ≥5 ng/mL (Fisher p=0.0004; OR 0.33; 95% CI 0.17–0.62). After multivariable adjustment, the aOR was 0.35 (95% CI 0.18–0.66; p=0.002; **Figure 3**). In an exploratory continuous-P4 model adjusted only for baseline covariates (no ΔP4 adjustment), each 1 ng/mL increase in pre-ET P4 was associated with an aOR of 1.19 for live birth (95% CI 1.04–1.35; p=0.009).

Among clinical pregnancies, miscarriage was 46.7% in the <5 subgroup, compared with 12.5% (5–7.49) and 5.7% (7.5–9.99). CPR was 38.5% below 5 ng/mL versus 48.1% at ≥5 ng/mL. These outcome components are interpreted in the Discussion.

### 3.5 Comparison with the no-rescue group

**Figure 4:**
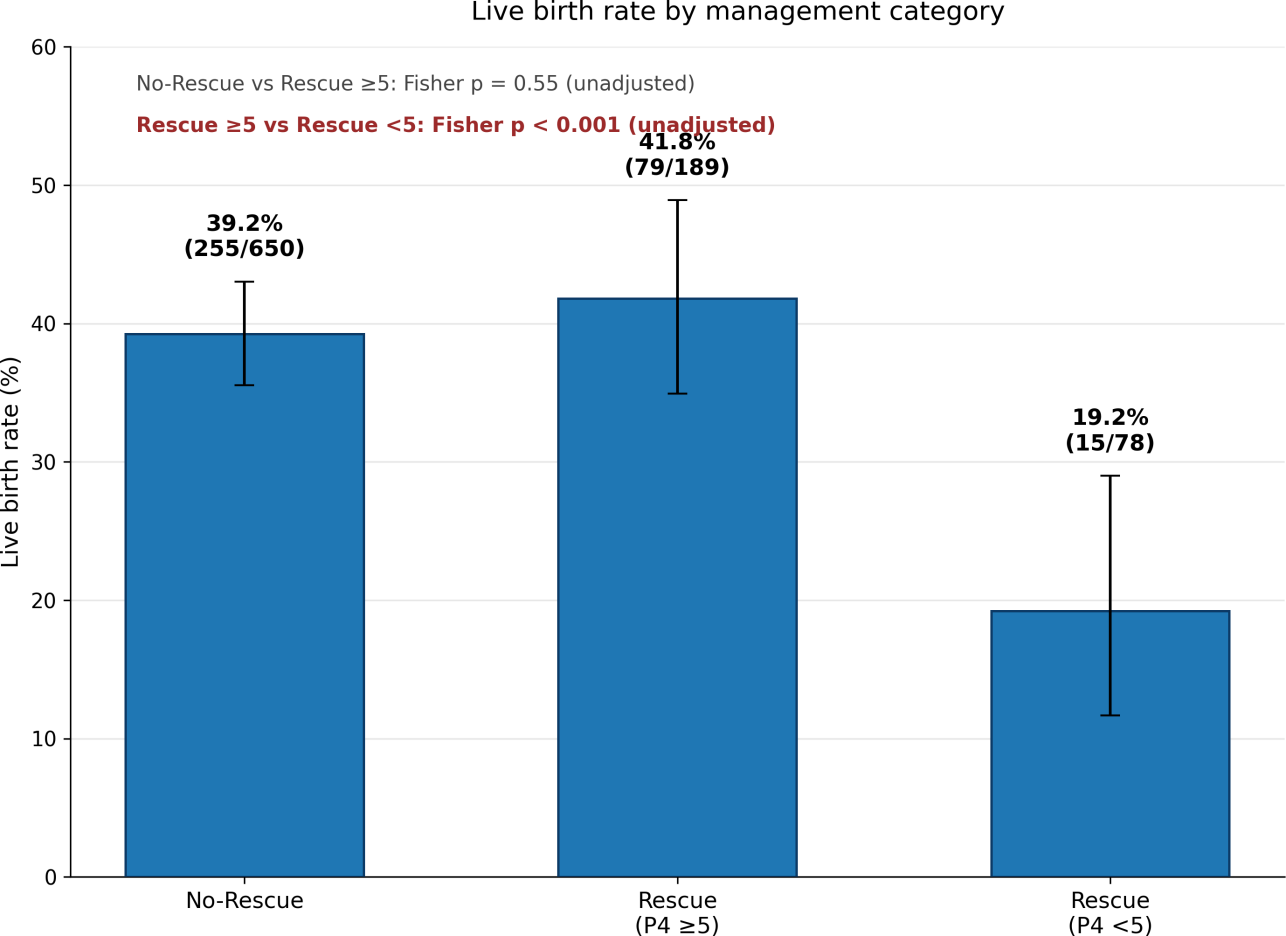
Live birth rate by three management categories: no-rescue, rescue ≥5 ng/mL, and Rescue <5 ng/mL. Bars show exact binomial 95% confidence intervals; unadjusted rates are shown, and no formal equivalence or causal rescue effect was tested.

Among Rescue patients with P4 ≥5 ng/mL, LBR was 41.8% (79/189), similar in unadjusted comparison to the No-Rescue group’s 39.2% (255/650; Fisher p=0.55; **Figure 4**). Formal equivalence testing was not performed. Because group membership was determined by rescue receipt rather than by random allocation, this comparison cannot isolate the effect of rescue or determine whether deferral would have improved outcomes. In the P4 <5 group, LBR was 19.2% (15/78). Within the borderline 9.1–9.9 ng/mL window where rescue was applied at clinician discretion, 21 cycles received rescue and 40 did not; LBR was numerically higher with rescue (12/21, 57.1%; 95% CI 36.5–75.5) than without (16/40, 40.0%; 95% CI 26.3–55.4; Fisher OR 2.00, p=0.28). Although underpowered, this small naturalistic comparison provides a limited within-cohort descriptive contrast at the upper boundary of the trigger threshold, not a causal estimate of rescue efficacy, and is interpreted cautiously in Discussion 4.6.

### 3.6 ROC and spline analyses

**Figure 5:**
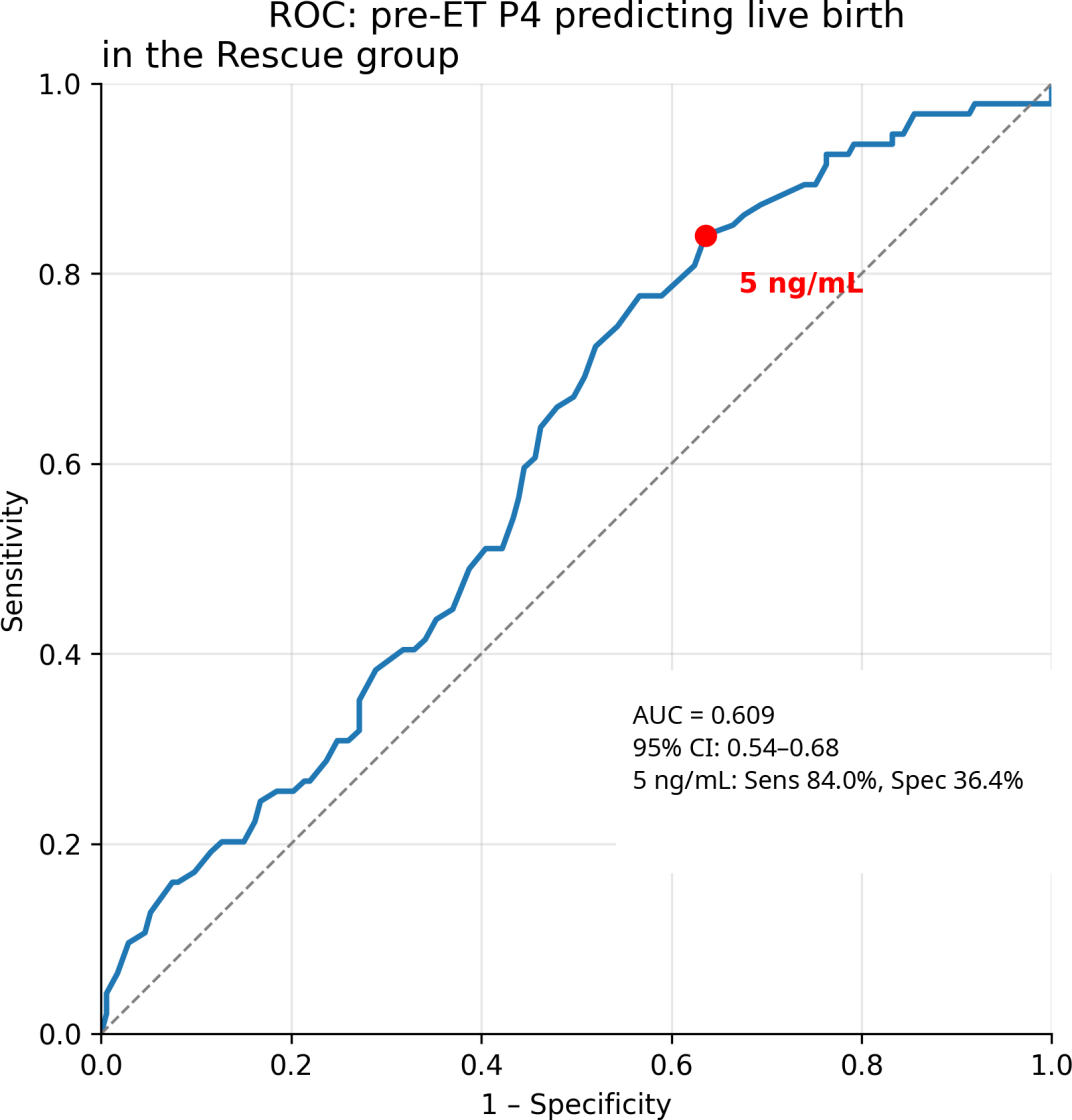
ROC curve for pre-transfer progesterone predicting live birth in the Rescue group. The red point marks the operating characteristics at the exploratory 5 ng/mL cutoff.

ROC analysis of pre-ET progesterone predicting LBR in the Rescue group yielded AUC 0.609 (95% CI 0.54–0.68; **Figure 5**), indicating modest individual-level discrimination. Youden’s optimal threshold was 5.3 ng/mL, close to the exploratory 5 ng/mL cutoff. At 5 ng/mL: sensitivity 84.0%, specificity 36.4%, NPV 80.8%. In 1000 bootstrap resamples, the median Youden threshold was 5.4 ng/mL (IQR 5.0–5.6; 2.5th–97.5th percentile 4.2–6.2). In out-of-bag validation of bootstrap-selected cutoffs, the median LBR difference above versus below the selected cutoff was 16.4 percentage points (IQR 11.1–21.8). These findings support internal stability only and do not establish a clinically actionable rule for deferral.

**Figure 6:**
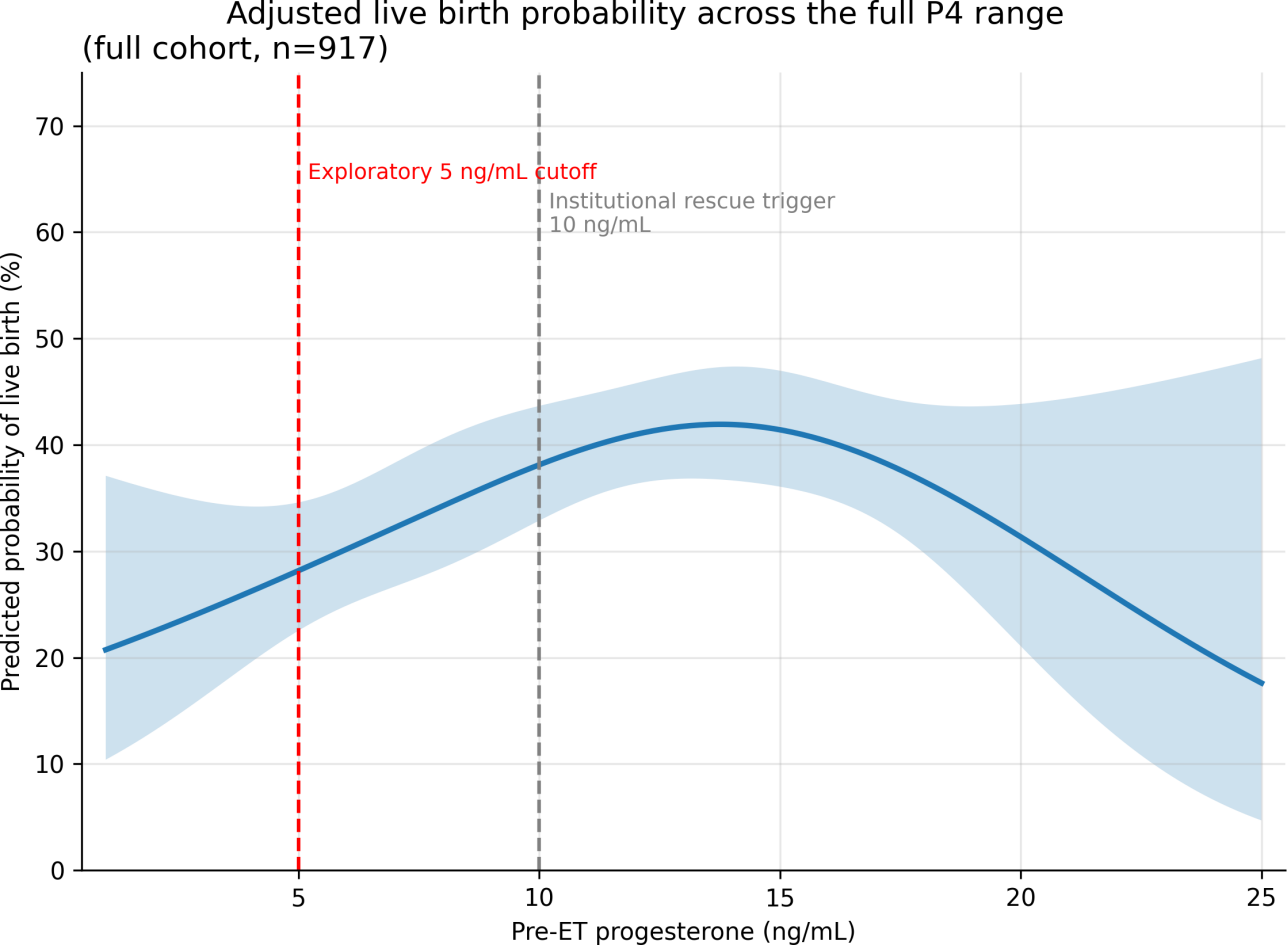
Restricted cubic spline of predicted live birth probability across the full progesterone range in the full cohort (n=917). The dashed red line marks the exploratory 5 ng/mL cutoff; the dashed gray line marks the 10 ng/mL rescue-initiation threshold.

**Figure 7:**
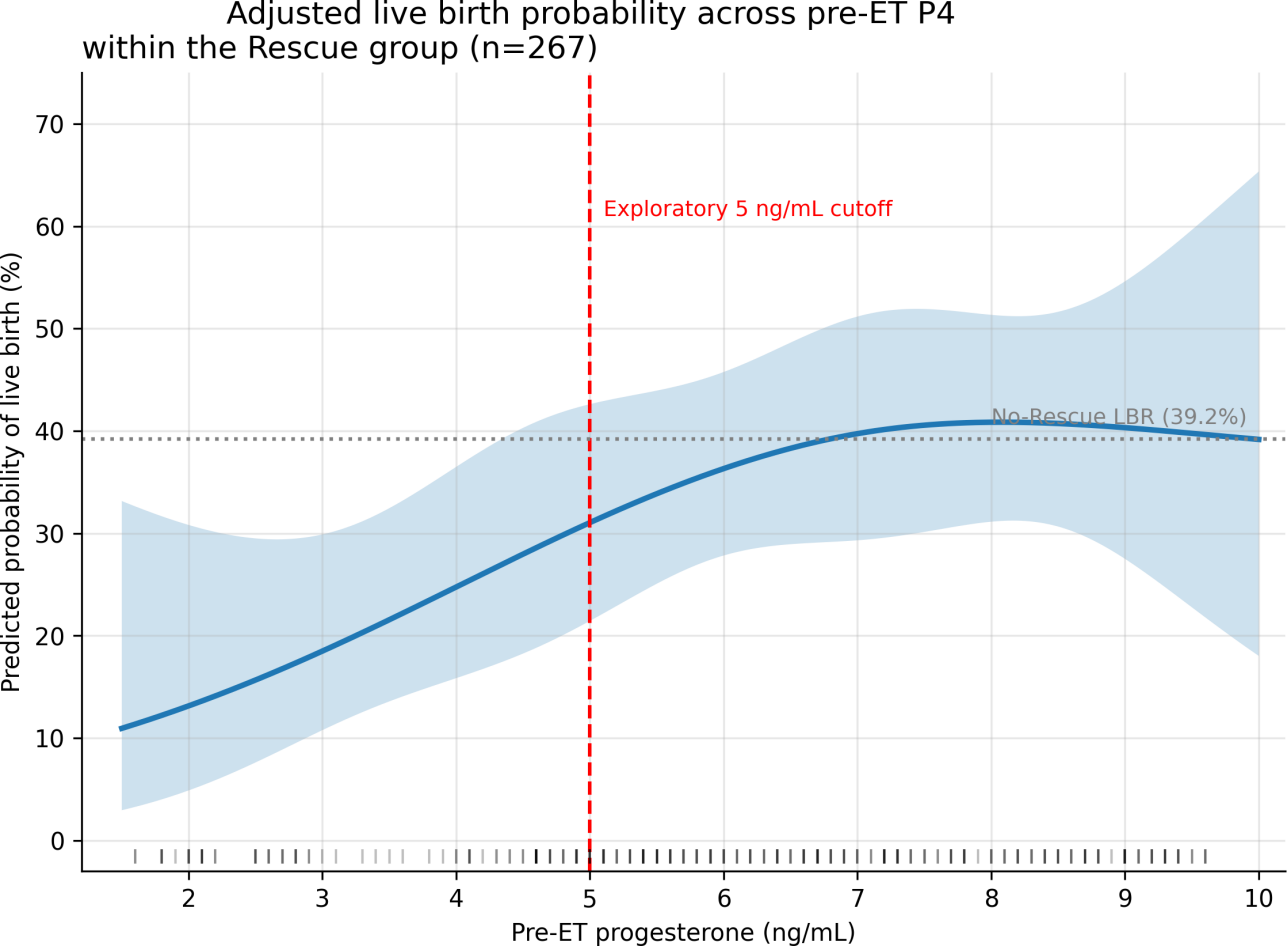
Restricted cubic spline of predicted live birth probability as a function of pre-transfer progesterone in the rescue group. The shaded band indicates the 95% confidence interval; the dotted blue line marks the No-Rescue live birth rate (39.2%).

RCS in the full cohort suggested nonlinearity (p=0.045), with LBR rising from very low P4 values and then plateauing (**Figure 6**). Within the Rescue group, RCS showed an overall P4 association (p=0.04) with a predominantly linear dose–response (nonlinearity p=0.39) and lower predicted LBR around the <5 ng/mL range (**Figure 7**).

### 3.7 Perinatal outcomes

Among 349 live births (255 No-Rescue, 94 Rescue), gestational age (39.0 ± 1.6 vs. 39.1 ± 1.8 weeks) and birth weight (3,301 ± 501 vs. 3,290 ± 473 g; 8 missing values) were comparable. Preterm birth, re-derived from GA <37 weeks, was 9.4% (No-Rescue) versus 6.4% (Rescue). SGA (1.6% vs. 0%), NICU (2.4% vs. 4.6%), GDM (1.2% vs. 0%), and hypertensive disorders (2.4% vs. 2.3%) did not differ significantly (**Table 5**). Cesarean delivery was higher in the Rescue group (11.8% vs. 4.7%; p=0.02), which may reflect chance variation or unmeasured obstetric confounding.

**Table 5:** Obstetric and neonatal outcomes among live births.

Group	n live births	GA, weeks	Birth weight, g	Preterm, %	SGA, %	NICU, %	GDM, %	HTN, %	Cesarean, %
No-Rescue	255	39.0 (1.6)	3301 (501)	9.4	1.6	2.4	1.2	2.4	4.7
Rescue	94	39.1 (1.8)	3290 (473)	6.4	0.0	4.6	0.0	2.3	11.8

Preterm birth was re-derived from gestational age <37 weeks. Birth weight was missing for 8 live births.

### 3.8 Sensitivity analyses

Sensitivity analyses showed a similar direction of association. Blastocyst-only (n=247 Rescue): LBR 21.4% (<5, n=70) vs. 41.2% (≥5, n=177; p=0.003). SET-only (n=216 Rescue): LBR 18.8% (<5, n=69) vs. 39.5% (≥5, n=147; p=0.003). These analyses do not address embryo aneuploidy because PGT-A data were unavailable.

## 4 Discussion

### 4.1 Principal findings

In this retrospective cohort of 917 HRT-FET cycles managed under a single-dose IM rescue protocol, pre-ET progesterone <5 ng/mL was associated with poor per-transfer outcomes after rescue: LBR was 19.2% with a miscarriage rate of 46.7% among clinical pregnancies, compared with 41.8% LBR above this cutoff. The finding should be interpreted as a candidate poor-prognosis signal specific to this protocol and assay, not as a validated rule for deferral. Because rescue was administered at clinician discretion rather than at random and the only naturalistic rescue versus no-rescue contrast was confined to a small borderline 9.1–9.9 ng/mL subset, the study cannot reliably determine whether rescue caused, improved, or failed to improve outcomes; and because no cycles were deferred, it cannot determine whether deferral would improve cumulative live birth rate.

### 4.2 Interpretation of the pregnancy-loss pattern

The LBR difference below 5 ng/mL appeared to involve both lower CPR (38.5% vs. 48.1%) and higher miscarriage among clinical pregnancies (46.7% vs. 9.9% above 5 ng/mL). This pattern is biologically plausible because inadequate progesterone exposure may impair decidualization, endometrial receptivity, and early pregnancy maintenance (6, 7). The serial trajectory raises a protocol-specific concern: although ET-day serum levels were clinically similar between groups, these were non-trough post-injection values and did not represent sustained exposure. Formal analysis confirmed a substantially larger ET-day to ET + 5 decline after rescue than in non-rescued cycles. At the same time, ET + 5 progesterone did not differ between the pre-ET <5 and ≥5 subgroups within Rescue and was not associated with LBR in exploratory analysis. Therefore, the observed poor-prognosis signal should not be interpreted as proof that any single post-transfer serum value determines implantation. Rather, very low pre-ET progesterone may mark an early pharmacokinetic or endometrial-exposure vulnerability under a single-dose rescue protocol, with the underlying mechanism unresolved by serum measurements alone. Causality cannot be inferred from these observational data, and the high miscarriage rate may also reflect embryo factors, including aneuploidy, because PGT-A was not performed.

### 4.3 Relationship to existing literature: initiation thresholds versus post-rescue prognosis

Previous studies have reported progesterone values used to identify patients who may need rescue or individualized luteal support, including thresholds around 8–13 ng/mL in non-rescued or pre-rescue settings (1, 10). These are rescue-initiation or progesterone-adequacy thresholds, not thresholds describing poor prognosis after rescue has already been given. Our <5 ng/mL finding addresses a different construct: prognosis after a single 50 mg IM rescue dose in patients who all proceeded to transfer. Direct numerical comparison between these constructs is therefore inappropriate. Lopez Marin et al. (2025) reported reduced efficacy of individualized luteal support in very low-progesterone patients (16), and studies of subcutaneous progesterone or dydrogesterone evaluate different routes, frequencies, and clinical decision points (13, 17–19). Collectively, the literature suggests that both the timing and intensity of rescue matter, but it does not establish a universal cutoff. Our data complement this literature by describing a low-P4 subgroup with poor outcomes under one single-dose protocol; prospective external validation is required before using this cutoff for clinical decisions.

### 4.4 Assay-specific threshold interpretation and cross-platform translation

The candidate <5 ng/mL signal reported in this study is specific to the Cobas e411 ECLIA platform and cannot be directly applied to other assay methods without recalibration. Published assay-comparison studies show platform-dependent bias: in the ESPRIT study, Elecsys^®^ Progesterone Gen III correlated closely with LC-MS/MS but showed a mean relative difference of 14.6% versus LC-MS/MS, while other immunoassay platforms showed different biases in IVF populations (14, 24). These data support assay-specific interpretation rather than direct transfer of a numerical cutoff.

**Table 6** presents estimated, non-validated threshold equivalents across commonly used assay platforms. These estimates are derived from published cross-platform comparisons and should be interpreted only as approximate guidance pending direct validation studies (14, 20, 21, 24).

**Table 6:** Estimated, non-validated progesterone equivalents for the candidate low-P4 poor-prognosis signal identified in this single-dose rescue protocol.

Assay platform	Manufacturer	Method	Estimated equivalent for candidate signal (ng/mL)	Interpretation	Reference
Cobas e411/Elecsys^®^ Progesterone III	Roche Diagnostics	ECLIA	5.0 (study platform)	Index assay used in this cohort; not externally validated as a deferral rule	(14)
LC-MS/MS	Multiple	Mass spectrometry	Approx. 4.0–4.75	Provisional research equivalent only; local validation required	(14, 24)
Architect i1000SR/i2000 progesterone	Abbott	CLIA	Platform-specific	Published IVF comparison showed assay-specific bias; do not apply Cobas cutoff directly	(24)
ADVIA Centaur progesterone/other Siemens platforms	Siemens Healthineers	CLIA	Platform-specific	Published IVF comparison showed assay-specific bias; split-sample validation required	(24)
Urine progesterone immunoassays	Platform-dependent	Immunoassay	Not directly convertible	Urine-to-serum and platform-specific calibration required	(21)

Conversion estimates are approximate and not validated. Centers should perform split-sample validation against Cobas e411 ECLIA or LC-MS/MS before applying any numerical cutoff clinically. Values are shown in ng/mL.

Centers using LC-MS/MS or alternative immunoassay platforms should not apply the 5 ng/mL cutoff directly. Instead, they should either: (1) conduct internal split-sample validation comparing their platform with Cobas e411 ECLIA, or (2) use an estimated LC-MS/MS equivalent of approximately 4.0–4.75 ng/mL as a provisional research reference pending validation. Clinical decisions should remain platform-specific and locally calibrated.

### 4.5 Single-dose versus repeated-dose rescue protocols

A critical limitation of the present study is that the rescue protocol consisted of a single 50 mg IM injection of progesterone. This may not represent the optimal rescue strategy. Our serial progesterone trajectory data demonstrate that single-dose rescue produced transient post-injection ET-day levels (16.4 ng/mL) that were clinically similar to No-Rescue values, but these non-trough values declined substantially to 7.8 ng/mL by ET + 5. This pharmacokinetic profile suggests that a single IM dose may provide insufficient sustained progesterone exposure during the implantation window for some patients.

A recent randomized controlled trial by Le et al. (2025) provides evidence for the importance of dosing frequency (15). In this RCT, patients with P4 <10 ng/mL on the day of FET were randomized to vaginal progesterone alone versus vaginal progesterone plus daily 50 mg IM progesterone. The daily IM supplementation group demonstrated higher clinical pregnancy rate (39.3% vs. 32.0%) and ongoing pregnancy rate (35.2% vs. 28.6%) (15). These findings differ from the present single-dose protocol and indicate that repeated dosing may overcome deficits that a single injection does not.

Whether repeated IM dosing, subcutaneous rescue (Yazbeck et al., 2025 (17)), or oral dydrogesterone (Mackens et al., 2023 (18); Cucchietti et al., 2025 (19)) could improve outcomes among patients with P4 <5 ng/mL remains unknown and requires prospective investigation. The present study can only describe outcomes after a single-dose IM rescue protocol; it cannot determine whether more intensive protocols would succeed at these very low baseline levels.

### 4.6 Clinical implications

These retrospective data support only a cautious, hypothesis-generating management framework. For P4 5–10 ng/mL, the observed outcomes are compatible with proceeding after single-dose IM rescue under this institutional protocol; in the small naturalistic comparison among borderline 9.1–9.9 ng/mL cycles, rescue was associated with a numerically higher LBR (57.1% vs 40.0%) but the 17-percentage-point difference did not reach statistical significance and the comparison is small, non-randomized, and clinician-selected. For P4 <5 ng/mL, clinicians should counsel patients that per-transfer prognosis was poor in this cohort (LBR ~19%, miscarriage ~47% among clinical pregnancies) and discuss options, including proceeding, deferring, or considering a more intensive rescue regimen where clinically appropriate. This framework should not be applied rigidly and does not prove that deferral improves cumulative outcomes.

### 4.7 Cumulative live-birth rate and deferral uncertainty

The present data cannot determine whether deferral improves cumulative live-birth rate (CLBR). All patients proceeded to transfer, so no deferred comparison group exists. Deferral may allow transfer in a later cycle with improved progesterone exposure, but it may also introduce treatment delay, additional monitoring, financial cost, patient distress, and possible embryo loss or attrition related to additional freeze-thaw procedures. Therefore, a low P4 value should not automatically mandate cancellation or deferral.

Clinical implementation should use shared decision-making that incorporates absolute per-transfer outcome estimates, embryo availability, patient age, previous treatment history, embryo quality or ploidy when available, tolerance for miscarriage risk, and personal preferences. Patients near the 5 ng/mL boundary may reasonably choose different options. Clinicians should present the observed LBR (~19%) and miscarriage rate (~47% among clinical pregnancies) alongside the uncertainty about CLBR and the absence of evidence that deferral is superior.

### 4.8 Strengths

Strengths include: (1) large single-center cohort (n=917) with consistent protocols over 7 years; (2) uniform single-dose rescue protocol (50 mg IM) and assay platform; (3) serial three-timepoint progesterone monitoring; (4) multiple exploratory analyses assessing the same association, including bootstrap internal stability analysis; (5) data quality verified against source patient records; (6) sensitivity analyses; (7) baseline balance for most clinical covariates; (8) Statistical code will be uploaded as **Supplementary File S4** for reproducibility; (9) STROBE compliance.

### 4.9 Limitations

First, the retrospective single-center design is subject to unmeasured confounding and cannot isolate the rescue effect. Group membership was determined by rescue receipt, which followed an institutional 10 ng/mL trigger threshold but was applied at clinician discretion at the boundary; the only within-cohort naturalistic comparison of rescue versus no rescue was confined to a small borderline 9.1–9.9 ng/mL subset (n=61) and is underpowered. Second, no cycles were deferred, so we cannot demonstrate that deferral improves CLBR. Third, the <5 ng/mL finding is assay-specific to Cobas e411 ECLIA and should be treated as an exploratory candidate signal rather than a validated clinical threshold. Fourth, the rescue protocol used a single 50 mg IM injection; this may not represent optimal rescue, and daily IM dosing may overcome deficits that single-dose rescue does not (15). Fifth, embryo quality was based on morphological grading without PGT-A. The higher miscarriage rate in the <5 ng/mL group could reflect insufficient progesterone exposure, embryo aneuploidy, or both; blastocyst-only analysis provides partial reassurance but does not resolve this confounding. Sixth, the study cannot distinguish low serum progesterone exposure from altered absorption or distribution, intrinsic endometrial progesterone responsiveness, or impaired progesterone receptivity, because endometrial progesterone receptor expression, molecular receptivity testing, and tissue-level progesterone exposure were not measured; the very low pre-ET P4 group is therefore mechanistically heterogeneous. Seventh, although the rescue protocol, assay platform, and clinical decision-making framework remained unchanged throughout 2017–2023, formal time-period sensitivity analyses were not performed because de-identified annual cycle timestamps were not available in the analytic dataset. Eighth, the ROC AUC of 0.609 indicates modest individual-level predictive ability, and bootstrap internal validation does not replace external validation. Ninth, despite re-checking patient records to verify data quality, residual undetected errors cannot be excluded. Tenth, cycles canceled before progesterone monitoring due to inadequate endometrial development are not included, which may limit generalizability.

### 4.10 Future directions

Future studies should include: (1) multicenter prospective external validation using standardized progesterone timing and assay-specific calibration; (2) split-sample comparison between Cobas e411 ECLIA, other immunoassays, and LC-MS/MS to establish local conversion factors; (3) randomized or target-trial emulation designs comparing rescue-and-proceed, intensified rescue, and deferral with CLBR as the primary endpoint; (4) dose-escalation studies evaluating daily IM rescue through the implantation window, as in Le et al. (15); (5) incorporation of PGT-A or embryo ploidy data to separate endometrial from embryonic causes of miscarriage; (6) assessment of endometrial progesterone responsiveness, progesterone receptor expression, or molecular receptivity in patients with very low pre-ET serum progesterone, to distinguish low circulating exposure from intrinsic endometrial unresponsiveness; and (7) time-stratified analyses in cohorts with dated cycles to examine temporal drift.

## 5 Conclusion

In 917 HRT-FET cycles managed with a single-dose 50 mg IM progesterone rescue protocol, pre-transfer progesterone <5 ng/mL was associated with lower LBR (19.2%) and high miscarriage among clinical pregnancies (46.7%). This finding is an exploratory, assay-specific poor-prognosis signal, not a validated rule for deferral. Because rescue was administered at clinician discretion rather than at random and no cycles were deferred, the study cannot reliably isolate the effect of rescue or show that deferral improves CLBR. The 5 ng/mL value is specific to Cobas e411 ECLIA and requires recalibration for other platforms. Prospective external validation and studies of repeated-dose rescue protocols are required before clinical implementation.

## Data Availability

The de-identified analytic dataset (**Supplementary Table S3**), complete statistical code (Other **Supplementary File 1**) and the complete statistical outputs (**Supplementary Table S2**) are provided as **Supplementary Material**. Further inquiries can be directed to the corresponding author.

## Supplementary material

Supplementary Table S1 STROBE checklist (provided as a separate Word document).

Supplementary Table S2 Complete statistical outputs workbook (All_Tables_v2.xlsx).

Supplementary Table S3 De-identified analytic dataset (Dataset_FINAL_CLEANED_v2.xlsx).
