# Supplementary material for: Serum progesterone levels for ‘rescue’ in hormone replacement FET: a retrospective cohort study of 917 cycles
Source: Front Endocrinol (Lausanne). 2026 Jun 1;17:1847028. doi: 10.3389/fendo.2026.1847028 (PMC13265284; doi:10.3389/fendo.2026.1847028)
Supplement: Supplementary Table S1 — STROBE checklist (provided as a separate Word document). [file DataSheet1.docx]

**STROBE checklist for cohort studies**

*Serum progesterone levels for rescue in HRT-FET*

| **Item** | **No.** | **Recommendation** | **Reported on** |
| --- | --- | --- | --- |
| Title and abstract | 1 | (a) Study design; (b) informative abstract | Title page, Abstract |
| Introduction |  |  |  |
| Background/rationale | 2 | Scientific background and rationale | §1, paragraphs 1–3 |
| Objectives | 3 | Specific objectives | §1, paragraph 4 |
| Methods |  |  |  |
| Study design | 4 | Key elements of study design | §2.1 |
| Setting | 5 | Setting, locations, dates | §2.1 (Jan 2017–Dec 2023, single center) |
| Participants | 6 | Eligibility, selection | §2.2 (first cycle per patient; 43 repeats excluded) |
| Variables | 7 | Outcomes, exposures, confounders | §2.5–2.6 |
| Data sources | 8 | Data sources and assessment | §2.4: Cobas e411 ECLIA, Elecsys Progesterone III (Roche), CV <5% |
| Bias | 9 | Efforts to address bias | §2.7 (data quality), §2.8 (adjusted models), §4.9 |
| Study size | 10 | How study size arrived at | §2.2 (all eligible; n=917) |
| Quantitative variables | 11 | Handling of quantitative variables | §2.6 (subgroups + binary); §2.8 (continuous + RCS) |
| Statistical methods | 12 | All statistical methods | §2.8 (regression, RCS, ROC, trend, sensitivity) |
| Results |  |  |  |
| Participants | 13 | Numbers at each stage; flow diagram | §3.1, Figure 1 |
| Descriptive data | 14 | Characteristics; missing data | Table 1; §2.7 (missing <5%; 8 BW missing) |
| Outcome data | 15 | Numbers of outcome events | Tables 3–5 |
| Main results | 16 | Unadjusted and adjusted estimates | §3.4 (Fisher + aOR); Table 4; Figure 5 |
| Other analyses | 17 | Subgroup, sensitivity | §3.6 (ROC, RCS); §3.8 (blastocyst, SET) |
| Discussion |  |  |  |
| Key results | 18 | Summarise key results | §4.1 |
| Limitations | 19 | Limitations, bias, precision | §4.9 (9 limitations) |
| Interpretation | 20 | Cautious interpretation | §4.1–4.4 |
| Generalisability | 21 | External validity | §4.4 (assay-specific); §4.6–4.7 (shared decision-making) |
| Other |  |  |  |
| Funding | 22 | Source of funding | Funding section (none) |
