# Supplementary material for: Serum progesterone levels for ‘rescue’ in hormone replacement FET: a retrospective cohort study of 917 cycles
Source: Front Endocrinol (Lausanne). 2026 Jun 1;17:1847028. doi: 10.3389/fendo.2026.1847028 (PMC13265284; doi:10.3389/fendo.2026.1847028)
Supplement: Supplementary Table S2 — Complete statistical outputs workbook (All_Tables_v2.xlsx). [file DataSheet2.docx]

**Supplementary Materials**

*Serum Progesterone Levels for ‘Rescue’ in Hormone Replacement FET: a retrospective cohort study of 917 cycles*

Goc G, Sukur YE

# Supplementary File Inventory

| **Item** | **Description** | **File name** |
| --- | --- | --- |
| Supplementary Table S1 | STROBE checklist | Supplementary Table S1. STROBE checklist.docx |
| Supplementary Table S2 | Complete statistical outputs workbook | Supplementary Table S2. Complete statistical outputs workbook.xlsx |
| Supplementary File S3 | De-identified analytic dataset | Supplementary File S3. De-identified analytic dataset.xlsx |

# Data corrections applied in version 2

Version 2 of the analytic dataset retains the previously validated corrections to clinical-pregnancy coding, embryos transferred, birth-weight data type, delivery-mode harmonization, and the day-5 progesterone decimal error.

In the final assembly pass, fields with internal inconsistencies were re-checked against the source patient records and corrected to match the source. Preterm birth was reconciled with gestational age <37 weeks among live births (41 records corrected); the 2 records with contradictory live-birth/miscarriage flags were verified against patient files and corrected to match the source; non-live-birth perinatal fields were cleared after source confirmation, except for one separately retained stillbirth at 32 weeks (PX1258).

Additionally, 31 records showed blastocyst counts exceeding fertilized-oocyte counts. The original patient laboratory records were re-checked, and fertilized (2PN), mature-oocyte (MII), and oocyte-retrieval counts were corrected to match the source documentation (93 total cell updates).

# Workbook contents (Supplementary Table S2)

- Table 1 — Baseline characteristics by study group
- Table 2 — Serial progesterone levels by group and timepoint
- Table 3 — Primary reproductive outcomes by study group
- Table 4 — Rescue-group subgroup analysis by pre-transfer progesterone
- Table 5 — Obstetric and neonatal outcomes among live births
- Table 6 — Estimated, non-validated assay-specific translation table
- Model Full — Multivariable model for the full cohort
- Model Rescue P4 — Rescue-only continuous progesterone model adjusted for baseline covariates only (no ΔP4; per Reviewer 3)
- Model Threshold — Exploratory Rescue-only <5 vs. ≥5 ng/mL model
- RCS Full — Restricted cubic spline predictions for the full cohort
- RCS Rescue — Restricted cubic spline predictions for the Rescue group

• Bootstrap Summary — Internal bootstrap stability analysis of the ROC/Youden-derived candidate cutoff

• Borderline 9.1–9.9 — Naturalistic sensitivity comparison of rescue versus no rescue at the trigger boundary

• Serial P4 Trajectory — Formal within-group and between-group evaluation of ET-day to ET+5 progesterone decline

• ET5 Sensitivity — ET+5 progesterone within the Rescue group: subgroup means by pre-ET <5 vs ≥5 ng/mL, univariable logistic regression of ET+5 P4 on live birth, and joint model adjusting for pre-ET <5 status (per Reviewer 3)

# Key confirmed numbers

Cohort: 917 unique patients (650 No-Rescue, 267 Rescue).

No-Rescue: CPR 45.8%, LBR 39.2% (255/650), miscarriage per clinical pregnancy 14.4%.

Rescue overall: CPR 45.3%, LBR 35.2% (94/267), miscarriage per clinical pregnancy 19.0%.

Rescue subgroup outcomes: <5.0 ng/mL, LBR 19.2% (15/78), miscarriage per clinical pregnancy 46.7%; 5.0–7.49 ng/mL, LBR 43.8% (49/112); 7.50–9.99 ng/mL, LBR 39.0% (30/77).

Exploratory binary cutoff comparison: Fisher p = 0.0004; adjusted OR 0.35 (95% CI 0.18–0.66), p = 0.002.

Rescue ≥5 ng/mL versus No-Rescue: 41.8% versus 39.2% live birth rate; formal equivalence was not tested.

Perinatal outcomes among live births: preterm birth 9.4% (24/255) in No-Rescue and 6.4% (6/94) in Rescue.

Reviewer 3 post-hoc analyses (manuscript Sections 2.8, 3.2, 3.4): Continuous pre-ET P4 model adjusted only for baseline covariates (no ΔP4 adjustment) yielded aOR 1.19 per 1 ng/mL (95% CI 1.04–1.35; p=0.009). ET-day to ET+5 progesterone declined by −8.59 ± 5.20 ng/mL in Rescue (95% CI −9.22 to −7.96; paired p<0.001) versus −3.09 ± 7.08 ng/mL in No-Rescue (95% CI −3.64 to −2.54); between-group difference in change was −5.50 ng/mL (95% CI −6.33 to −4.67; Welch p<0.001). Within Rescue, ET+5 P4 was similar in pre-ET <5 versus ≥5 ng/mL subgroups (7.71 ± 2.92 vs. 7.86 ± 3.20 ng/mL; Welch p=0.72), and ET+5 P4 was not associated with LBR (OR per 1 ng/mL 0.98; 95% CI 0.90–1.06; p=0.57).

# Assay note

The candidate 5 ng/mL poor-prognosis signal reported in this manuscript is specific to the Cobas e411 ECLIA platform (Roche Elecsys® Progesterone III). Cross-platform equivalents shown in Table 6 are provisional, non-validated translation estimates derived from published immunoassay-to-LC-MS/MS comparisons and should not be used without local validation.
